# Public attitudes towards gambling product harm and harm reduction strategies: an online study of 16–88 year olds in Victoria, Australia

**DOI:** 10.1186/s12954-017-0173-y

**Published:** 2017-07-25

**Authors:** Samantha L. Thomas, Melanie Randle, Amy Bestman, Hannah Pitt, Steven J. Bowe, Sean Cowlishaw, Mike Daube

**Affiliations:** 1 Centre for Population Health Research, School of Health and Social Development, Faculty of Health, Deakin University, Geelong, Australia; 2 School of Management, Operations and Marketing, Faculty of Business, University of Wollongong, Wollongong, Australia; 3 Deakin Biostatistics Unit, Faculty of Health, Deakin University, Geelong, Australia; 4 School of Social and Community Health, University of Bristol, Bristol, UK; 5 Faculty of Health Science, Curtin University, Bentley, Australia

## Abstract

**Background:**

Gambling has quickly emerged as an important global public health issue. With new technologies used to develop high intensity gambling products and promotions aimed at driving consumption, public health organisations and researchers, community groups, and politicians have argued for a range of regulatory and education measures aimed at reducing gambling harm. However, there has been limited research seeking to understand community perceptions of the harms associated with gambling products and environments, and the level of community support for strategies designed to prevent and reduce gambling harm.

**Methods:**

An online study of 500 adolescents and adults (aged 16 and over) was conducted with a representative sample (by age and gender) of individuals who were current residents in the state of Victoria, Australia. Participants were asked a range of questions about their own gambling behaviours, with the Problem Gambling Severity Index (PGSI) used as a measure of problem gambling. Participants were asked about their perceptions of harms associated with electronic gambling machines (EGMs), sports betting, horse betting, and casino gambling. They were also asked about the extent to which they agreed or disagreed with gambling harm reduction strategies related to marketing and promotions, restrictions on gambling products and venues, and public education campaigns. Quantitative data were analysed using descriptive statistics and paired *t* tests, with thematic analysis used to interpret qualitative responses to open-ended questionnaire items.

**Results:**

More than one third (*n* = 201, 40.2%) of participants were at risk of experiencing some level of harm from gambling (PGSI ≥ 1), with 83 participants (16.6%) recording scores that indicated problem gambling (PGSI ≥ 8). One in five participants gambled on EGMs at least monthly (*n* = 100, 20.0%). Those who gambled on sports did so frequently, with nearly 1 in 5 gambling on sport at least once a month (*n* = 87, 17.4%). Over half of the sample rated casino gambling and EGMs as very harmful, while one third rated these forms of gambling as extremely harmful. Over one third of the sample rated horse and sports betting as very harmful, with one in five rating these products as extremely harmful. There was strong agreement with the need to ban gambling advertising during children’s viewing hours, during sporting matches and at sporting venues. The majority of participants agreed with reducing and restricting EGMs, and providing more public education for both adults and children about the negative consequences from gambling.

**Conclusions:**

The findings suggest a strong perception in the Victorian community that gambling products are harmful. While governments have been reluctant to implement a comprehensive approach to reducing gambling harm, this study reveals significant community support for a range of harm reduction and prevention measures associated with gambling products. Public health practitioners can use this evidence in advocating for a comprehensive public health approach to reducing the harms associated with gambling promotions and products.

## Background

Over the last two decades there have been significant shifts towards more liberalised gambling regulatory frameworks in countries such as Australia, Great Britain, New Zealand, and Ireland, which have increased the availability and accessibility of high intensity gambling products in community settings [1, 2]. Some argue that governments have been complicit in supporting the interests of the gambling industry [1], with changes in government policy enabling the proliferation of high intensity gambling in community environments [3–5]. More recently, the liberalisation of gambling has led to the legalisation of more pervasive forms of gambling such as online sports betting. These policy shifts have occurred in parallel with the development of new technologies and higher intensity products [6], the use of both traditional and social media platforms to promote and incentivise product use [7], and strategies aimed at aligning gambling with culturally valued activities such as sport [8]. While there are a number of proposed regulatory mechanisms that would reduce the harms associated with gambling products [9, 10], governments have been largely unwilling to enact a comprehensive public health approach to gambling, as applied in other areas such as tobacco [11]. Government regulatory and education efforts steadfastly focus on individualised responsibility frameworks to minimise the harms associated with ‘problem gambling’ [12, 13], which place few constraints on commercial activities and permit continued growth in both industry and government revenues (the latter through taxation).

There is increasing community dissatisfaction with the proliferation of gambling products within community spaces, and community opposition to the expansion of gambling in these spaces [14]. This raises an ethical tension for governments between the revenue that they may make from gambling, and being “mindful of the parameters of what the public regard as acceptable” when considering their policy responses to gambling [pg. 146] [15]. Public health practitioners have highlighted the importance of understanding public attitudes towards policy, particularly given strategies used by harmful consumption industries to influence public attitudes towards products [16]. For example, community surveys were regularly used during campaigns on tobacco to influence media advocacy strategies, to convince policy-makers of the need for regulatory change, and to assess the impact of denormalisation strategies on the attitudes of young people [17].

Despite the broad political and community debate about gambling harm, relatively few studies have sought to understand community attitudes towards gambling, gambling products, and the regulation of gambling. Studies have utilised the Attitudes Towards Gambling Scale (ATGS) [18] with some finding that attitudes towards gambling have become more positive in recent years [19], while other studies indicate that adults have moderately negative attitudes towards gambling and are supportive of gambling reform [15, 20]. Community surveys in Australia have consistently demonstrated community concern about gambling and support for regulatory reform of gambling products [15, 21, 22]. For example, McAllister [2014] analysed data from a 2012 national survey, and found strong support for people nominating bet limits before they gamble (75.3%), and for gambling to be more tightly controlled (73.5%) [15]. However, half of participants also agreed that gambling activities were advertised responsibly (50.0%). This is perhaps not surprising given that data was collected prior to the significant surge of gambling advertising within sport in Australia, with gambling (and in particular sports betting) advertising spend increasing by 160% between 2011 and 2015 [23]. Most recently, a question within a national opinion poll conducted by the Foundation for Alcohol Research and Education found that 73% of adults agreed that gambling advertising should be phased out during televised sporting broadcasts [24].

While each of the above studies provides important information about public attitudes towards gambling, no studies to date have examined adolescent and adult perceptions of the harms associated with different forms of gambling. Further, there is limited information regarding public support for the range of possible regulatory and policy approaches that would contribute towards a comprehensive public health approach to gambling harm. The present study aims to address this gap by exploring community attitudes towards different forms of gambling, and strategies that may reduce gambling harm. Specific objectives were to:

1. Explore how Victorian adolescents and adults attribute harm to different types of gambling activities; and
2. Examine the extent to which Victorian adolescents and adults support the introduction of strategies aimed at reducing the harms associated with gambling.

## Methods

We used an online panel survey to explore the attitudes of 500 Australian residents. The data presented in this paper was part of a broader study which aimed to understand community attitudes towards the normalisation of gambling and gambling products. Approval for the study was obtained from Deakin University Human Research Ethics Committee.

### Recruitment and sampling

Recruitment and data collection occurred in March 2017. An online panel company was used to recruit participants according to the sample specifications. This method enabled us to obtain a sample that matched the Victorian community by age and gender within time and budgetary constraints. While evidence indicates that online studies produce less incomplete data and are as representative of population samples as would be found in, for example, a postal survey [25], they can over represent individuals who have experienced gambling harm [26]. Individuals voluntarily register with online panel companies and receive points for completing surveys. The researchers programmed the survey and hosted the data collection using Qualtrics survey software. Participants were provided with a participant information sheet before agreeing to complete the survey. Sample quotas were set using Australian Bureau of Statistics (ABS) population figures for Victoria [27]. Eligible participants were aged 16 years or over, residents of the state of Victoria (verified by postcode), and had read the participant information sheet and provided consent to participate.

### Measures

#### Personal information

Socio-demographic questions asked about age, sex, postcode, education, and employment status. Participants were also asked questions about gambling behaviours. They were initially asked how frequently in the previous 12 months they had participated in four products of interest for this study (EGMs, sports betting, horse betting, and casino gambling), as well as other forms of gambling. Participants who stated that they had not participated in any form of gambling were classified as ‘non-gamblers’. All participants, including non-gamblers completed the Problem Gambling Severity Index (PGSI), a 9-item survey measure of problematic gambling [28]. A score was calculated to cluster participants according to interpretive categories; non-problem gambling (score of 0), low risk gambling (scores 1–2), moderate risk gambling (scores 3–7), or problem gambling (scores of 8–27). Postcode data was used to determine Socio-Economic Indexes for Areas (SEIFA) status through the measurement of Index of Relative Socio-Economic Advantage and Disadvantage. This is an area level measure that ranges from 1 (which reflects the lowest scoring 10% of areas) to 10 (representing the highest 10% of areas) [29, 30].

#### Perceptions of harm

We developed questions which aimed to determine perceptions of the degree of harm associated with four of the most harmful forms of gambling in Australia in terms of money lost: EGMs, sports betting, horse betting, and casino gambling. Participants rated how harmful they perceived each form of gambling to be based on a continuous sliding scale from 0 (‘not harmful at all’) to 100 (‘extremely harmful’). For the product that they perceived to be the most harmful, participants were asked an open response question about why they thought this product was harmful. Items used colloquial language for gambling products. For example, instead of the term electronic gambling machine, we used the term ‘pokies’, and rather than wagering we used the term ‘betting’.

#### Support for regulatory action

Participants were presented with a set of proposed regulatory actions which may be influential in reducing gambling harm. These related to the regulation of gambling advertising, increased regulation of EGMs, the prohibition of new casinos, credit-based gambling, and community education strategies. This list of questions were developed to capture attitudes towards some harm minimisation strategies that have been regularly discussed by governments and community organisations. We also included questions about responses to education initiatives which are part of a comprehensive public health approach to harm minimisation. Participants were asked to rate how much they agreed or disagreed with these statements on a 4-point bipolar scale including ‘strongly disagree’, ‘disagree’, ‘agree’, and ‘strongly agree’. Because we were asking participants to consider harm minimisation responses which may ultimately inform policy, we wanted participants to carefully consider and make a choice about each statement, rather than ticking a ‘neither agree nor disagree’ or ‘neutral’ box. Further, empirical studies have shown that inclusion of a neither agree nor disagree option at the centre of an agreement scale can often be misinterpreted by participants and either used as a ‘don’t know’ option or to reject the item’s assumptions [31, 32].

### Data collection

The survey was piloted before the data collection commenced: first with personal networks that included people who had experienced harms from gambling to ensure content validity of measures, and second with *n* = 32 people registered with the online panel company to check for technical errors with the online survey. Following this ‘soft launch’, the survey was revised and the (open-ended) qualitative questions relating to reasons for perceptions of harm were added. The initial 32 participants in the pilot phase were excluded from the final analysis. A total of 2611 people accessed the survey over a 2-week period, however, many potential participants were screened out because age and gender quotas had been filled. Individuals were also screened out if they did not consent to participate (*n* = 140), stated that they were under 16 years old (*n* = 4), or stated that they were not currently a Victorian resident (*n* = 12). We carefully examined the data and excluded 88 individuals for whom there was missing or unreliable data. For example, we removed one participant who indicated that she was 16 years old, but had 4 children under 16 years including a 16 year old. We also excluded a participant who filled in the survey as a 29-year-old male, but wrote that the researchers should disregard the responses as she was actually a 64-year-old female who wanted to see the questions that were asked. The online panel company was contacted, and replacements were provided for these individuals (matched by age group and gender). The result was a final sample of 500 participants who completed the full survey.

### Data analysis

The data were downloaded from Qualtrics to IBM Statistical Program for Social Sciences (SPSS) 22.0 for data cleaning, and was then transferred to Stata 14.0 (Stata Corp) for subsequent analysis. Sample characteristics such as gender, age, occupation, education, and SEIFA have been presented using percentages. Age was presented as continuous (with a mean and standard deviation) and was also categorised into age groups (16–24, 25–34, 35–44, 45–54, 55–64, and 65+). SEIFA was determined by deciles allocated by the ABS and were then collapsed into 3 categories (1–3, 4–7, 8–10). Occupation and education status were based on ABS classifications. We cross-checked responses of participants who indicated that they had not gambled in the previous 12 months against PGSI responses, and identified 4 participants who were classified as gamblers (low risk gambling = 1, moderate risk gambling = 1, problem gambling = 2). We clustered participants into five categories—non-gamblers, non-problem gambling, low risk gambling, moderate risk gambling, and problem gambling, with the non-gamblers who had indicated that they were still experiencing harm shifted into the appropriate categories. Based on ratings out of 100 for harm the research team agreed on arbitrary descriptors based on cut-off scores. Ratings of 50-74 indicated perceptions that products were ‘harmful’, scores of 75–100 indicated perceptions that products were ‘very harmful’, and ratings of 90–100 indicated perceptions that products were ‘extremely harmful’. Paired *t* tests were performed to compare mean level of perception of harm (0–100 scales) associated with gambling products and environments. Frequency counts from the 4-point bipolar scale were used to determine level of agreement with each statement about support for gambling harm reduction strategies. Results that refer to overall agreement report the combined totals for agree and strongly agree categories.

For the qualitative responses regarding the product that was perceived as most harmful, we conducted a basic thematic analysis to determine the key themes relating to perceptions of such harms. ST, HP, and AB read and re-read participant responses to group similarities and differences between responses. We then discussed these themes as a group and re-read responses to ensure that themes were consistent with the data as a whole. In reporting the data, we have corrected minor typographical errors in participants’ written responses.

## Results

### Sample description

Table 1 provides the socio-demographic and gambling characteristics of the sample, which was proportionally representative of the Victorian population by age and gender. Just over half of participants were females (*n* = 255, 51.0%), with a mean age of 44.9 years (ranging from 16 to 88 years, s.d. = 17.7). Fifteen people were under the age of 18 in this study. Just under half of these reported that they had gambled in the last month (*n* = 6, 40%). Over half of the total sample were employed in full-time, part-time, or casual work (*n* = 297, 59.4%), with one in five participants retired (*n* = 102, 20.4%). The vast majority of the sample completed year 12 or had an advanced level of education beyond high school (*n* = 441, 88.2%), with a similar proportion of the sample being from medium to high areas of socio-economic advantage (*n* = 427, 85.4%). More than one third (*n* = 201, 40.2%) scored ≥ 1 on the PGSI and were at some level of risk of harms from gambling, with 83 (16.6%) participants receiving a score indicating problem gambling (PGSI ≥ 8). One participant under the age of 18 scored as a low risk gambler (PGSI 1–2), and one participant under the age of 18 scored as a problem gambler (PGSI ≥ 8).

**Table 1:** Socio-demographic and gambling characteristics

	*n* = 500 (%)
Gender	
Male	245 (49.0)
Female	255 (51.0)
Age	
16–24	82 (16.4)
25–34	94 (18.8)
35–44	85 (17.0)
45–54	80 (16.0)
55–64	69 (13.8)
65 or older	90 (18.0)
Occupation	
Working full-time	193 (38.6)
Working part-time or casually	104 (20.8)
Unemployed but looking for work	23 (4.6)
Homemaker	32 (6.4)
Retired	102 (20.4)
Full-time student	41 (8.2)
Other	5 (1.0)
Education	
Below year 10	10 (2.0)
Year 10	49 (9.8)
Year 12	102 (20.4)
Certificate I, II, III, IV	73 (14.6)
Diploma/advanced diploma	63 (12.6)
Bachelors degree	131 (26.2)
Graduate diploma/graduate certificate	22 (4.4)
Postgraduate degree	50 (10.0)
SEIFA decile*	
1–3	71 (14.2)
4–7	203 (40.8)
8–10	224 (45.0)
PGSI	
Non-gambler	96 (19.2)
Non-problem gambling	203 (40.6)
Low risk gambling	59 (11.8)
Moderate risk gambling	59 (11.8)
Problem gambling	83 (16.6)

*n = 498 due to 2 postcodes not having a SEIFA determined decile allocated by the Australian Bureau of Statistics

Participants were asked how frequently they had engaged in different forms of gambling in the previous 12 months (Table 2). One in five participants had gambled on EGMs at least monthly (*n* = 100, 20.0%), with just under half the sample never gambling on EGMs in the past 12 months (*n* = 235, 47.0%). Over half of the sample had never gambled at a casino (*n* = 285, 57.0%), or bet on horses (*n* = 259, 51.8%), while nearly two thirds had not bet on sports in the previous year (*n* = 318, 63.6%). However, those who did gamble on sports did so frequently, with nearly 1 in 5 of all participants gambling on sports at least once a month (*n* = 87, 17.4%).

**Table 2:** Frequency of product use

	Never	Less than once a month	1–3 times per month	Weekly	More than once a week
	*n* (%)	*n* (%)	*n* (%)	*n* (%)	*n* (%)
EGMs	235 (47.0)	165 (33.0)	52 (10.4)	30 (6.0)	18 (3.6)
Casinos	285 (57.0)	141 (28.2)	39 (7.8)	22 (4.4)	13 (2.6)
Horse betting	259 (51.8)	159 (31.8)	39 (7.8)	20 (4.0)	23 (4.6)
Sports betting	318 (63.6)	95 (19.0)	42 (8.4)	23 (4.6)	22 (4.4)
Other*	166 (33.2)	127 (25.4)	74 (14.8)	107 (21.4)	26 (5.2)

*Other included lotteries, buying scratch tickets (scratchies), Keno, raffles, bingo, or dog racing

### Perception of harm

Table 3 identifies the *degree* of harm perceived to be associated with different forms of gambling (ratings out of 100). While participants perceived that the gambling associated with all products was harmful to some degree, the greatest harm was attributed to casino gambling and EGMs. The mean level of perceived harm associated with casinos was significantly higher than for horse betting (p < 0.0001) and sports betting (p < 0.0001). Similarly, the perceived harm associated with EGMs was significantly higher than for horse betting (p < 0.0001) and sports betting (p < 0.0001). Statistically significant pairwise differences are indicated by 95% confidence intervals (CIs) that do not overlap (see Table 3).

**Table 3:** Perceptions of harms associated with gambling products

	Mean	SE*	95% CI**	*N* (%) scored 75 and over ‘very harmful’	*N* (%) scored 90 and over ‘extremely harmful’
Casinos (*n* = 500)	75.55	1.06	(73.47, 77.64)	306 (61.2)	191 (38.2)
EGMs (*n* = 500)	74.65	1.07	(72.55, 76.74)	294 (58.8)	178 (35.6)
Horse betting (*n* = 500)	66.73	1.06	(64.64, 68.81)	227 (45.4)	98 (19.6)
Sports betting (*n* = 500)	66.48	1.10	(64.32, 68.63)	227 (45.4)	105 (21.0)

*SE = standard error

**95% CI = 95% confidence interval

Qualitative data collected in the open responses to this question provided further insight into the products perceived as most harmful.

#### Casino gambling

Participants who perceived that casino gambling was most harmful described the seductive nature of the venue. For example, some participants suggested that casinos portrayed sophistication while hiding the harm that occurred within venues:

Casinos give an unwarranted air of sophistication and expectation of large winnings which draw in the gullible*—*Male, 65 years and over, non-problem gambling.

They suggested that design features of casinos were key contributors to harm. Some participants talked about having “no concept of time” and that the environment encouraged gambling more than you normally would:

The atmosphere inside a casino makes it easy to get lost in time and keep on spending. The Las Vegas effect, the bright lights make it more fun—Female, 55–64 years, non-problem gambling.

The casino is a place that you can get lost in time. It is easy to gamble more than you intended and stay longer than planned*—*Female, 55–64 years, low risk gambling.

Some participants perceived that casinos were harmful because people could lose more money in these venues, as there were multiple different types of gambling in one place.

Gambling at a casino can result in losing way more money—Female, 25–34 years, non-problem gambling.

Some also commented on the particularly harmful outcomes of casino-based gambling:

People often use all the money they have to gamble at the casino–leaving them in poverty—Female, 16–24 years, non-gambler.

#### EGMs

Participants who perceived that EGMs were most harmful described various factors associated with such harm. The first related to perceptions of EGMs as deceptive or exploitative. For example, EGMs were described as being ‘rigged’ and creating unrealistic perceptions that you could win. Participants often commented on the features of machines which were described as “alluring, deceitful and untrustworthy”, “designed to make people lose money”, “anti-social”, “mindless”, and “addictive”. Some participants specifically commented that the design features of machines led to addiction and placed profits over the welfare of people:

Pokies are extremely harmful as they’re set to be profitable to their owner, yet allow the player to win just often enough to keep them hooked. They effectively encourage addictive behaviour—Male, 35–44 years, non-problem gambling.

Pokies. The companies make them colourful with bright lights and music to attract people to play them, hoping they will win lots of money—female, 35–44 years, low risk gambling.

A 35–44 year-old male with non-problem gambling stated that “Pokies are designed to key in on addictions”, while a 55–64-year-old male who was classified as a problem gambler stated that “pokies are the most harmful because they induce you to chase your losses. They do this (I believe) through near misses which encourage the player to keep going”. Some participants also commented that the government was complicit in the harm that was caused from EGMs because of their reliance on taxation revenue from losses:

I think pokies are the most harmful because they are designed to not let you win. At the press of a button your money is gone. They’re designed to get you in, get you hooked. And they don’t care about the damage they are doing and neither does the government because it benefits from regular people losing their money—Female, 25–34 years, non-problem gambling.

Others commented that a significant contributing factor associated with EGM harm was that they created a perception that they were not risky products.

Poker machines are very addictive and ostensibly seem to only carry a small risk because the ante can be as small as 1 cent, though a 1 cent machine can still cost a couple of dollars per spin, and several dollars per minute*—*Male, 45–54 years, moderate risk gambling.

Some participants also referred to the accessibility and availability of EGMs in community environments. They suggested that the harms associated with EGMs were related to the saturation of EGMs in communities, “pokies are everywhere”, which were ultimately designed to “make people lose money and owners make money.” Others commented that it was too easy for individuals to “just sit there for hours at a time” and “lose large amounts of money in a short time”. Participants described the vulnerability of certain sub-groups within the community to EGMs:

Because pokies are designed to make you lose money, and the people who are losing the money on them are the most vulnerable (seniors who already have not a lot of money)—Male, 25–35 years, non-problem gambling.

#### Horse betting and sports betting

Individuals who perceived that either horse or sports betting were most harmful referred to the multiple markets offered by online betting providers, the constant availability of opportunities to gamble, that it was easy to lose financial control while betting on apps, and the role of marketing in the normalisation of sports betting.

Many participants wrote negative comments about television advertising for online gambling companies, with one participant with moderate risk gambling behaviours commenting on the “constant barrage of advertising and sponsorship”. Participants also described the role of advertising in the normalisation of sports betting. For example, one participant described how excessive advertising created a perception that “everyone is doing it”, while another stated that the “push to make (sports betting) normal is insidious”.

Because so many are pushed to think that everyone must be interested in sports (hint: Not everyone is but the peer pressure is EXTREME) and the advertising people are capitalising on that. Mind you, pokies are only just behind—Female, 65 years and over, non-problem gambling.

Some participants described that horse racing was an integral part of Australian culture, and that it was almost considered ‘un-Australian’ not to bet on horses at certain times of the year, particularly on the Melbourne Cup horse race.

Some also described that the biggest harm posed by sports betting was that it ruined people’s enjoyment of sport, or that they believed that sport was compromised because of increased gambling options and markets. One participant stated that it changed the ‘notion of sport’ from an activity that was related to physical activity and team-based behaviour, to an activity that was implicitly aligned with gambling. For example, the following participant stated:

Sports should be for participation and engagement and health, not for financial gain or loss—Male, 45–54 years, non-gambler.

### Level of agreement and disagreement with proposed gambling harm reduction strategies

**Fig. 1:**
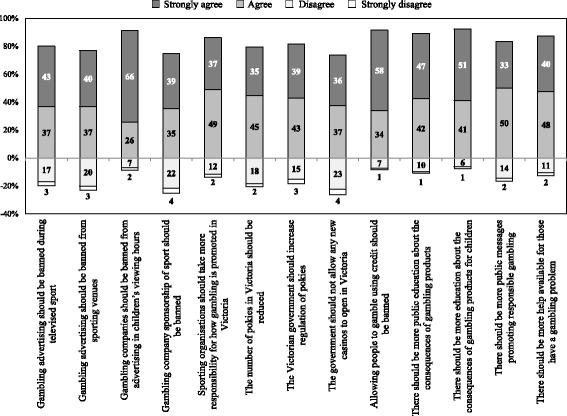
Level of agreement and disagreement with proposed harm reduction strategies

Participants were asked about the extent to which they supported a range of strategies aimed at reducing gambling harm (Fig. 1). The majority of participants agreed or strongly agreed with a complete ban on gambling promotions from televised sport (*n* = 400, 80.0%) and sporting venues (*n* = 385, 77.0%). More than 90% of participants also agreed or strongly agreed with a proposed ban on gambling advertising during children’s viewing hours (*n* = 457, 91.4%), with nearly two thirds of the sample (*n* = 329, 65.8%) indicating strong agreement. The large majority of participants (*n* = 431, 86.2%) agreed or strongly agreed that sporting organisations should take more responsibility for how gambling is promoted.

There were high levels of agreement with policies aimed at reducing and restricting the number of venues which offer opportunities to gamble. The majority of participants agreed or strongly agreed that the number of EGMs should be reduced (*n* = 397, 79.4%), with over one third strongly agreeing (*n* = 173, 34.6%). More than 80% agreed or strongly agreed with increased regulation of EGMs (*n* = 407, 81.6%), with over one third strongly agreeing (*n* = 193, 38.6%).

Finally, there was also strong agreement with proposals for increased public education about the harms associated with gambling. Most participants either agreed or strongly agreed that there should be more education about the consequences associated with gambling products (*n* = 446, 89.2%), and increased messages about responsible gambling (*n* = 416, 83.2%). The vast majority of participants agreed or strongly agreed with increased education for children about the consequences of gambling (*n* = 462, 92.4%), with over half of all participants strongly agreeing with this statement (*n* = 257, 51.4%).

## Discussion

This study aimed to investigate community perceptions of the harms associated with different gambling products, and levels of support for strategies that may reduce the harms associated with these products. This study raises a number of points for discussion.

First are the community perceptions of harm associated with gambling products. Participants perceived that the greatest harm was associated with casino gambling and EGMs, with the vast majority of participants indicating that these were either very or extremely harmful. Qualitative data revealed that some participants had some level of awareness that EGMs contained features that could be misleading or addictive. This was surprising given that public education does not generally focus on the harmful features of EGMs, and instead gives messages about help seeking or ‘responsible’ choices. This may suggest that information about EGMs is coming from other sources—for example, via news media reports or personal experiences of harm. However, despite a perception that EGMs were harmful, one in five participants reported gambling on EGMs at least monthly. This may suggest that perceptions of harm do not necessarily translate into behavioural choices. Fewer participants perceived that sports or horse betting were harmful products, which could be for several reasons. For example, horse racing is socially accepted in Victoria (with a public holiday for the Melbourne Cup horse race), while racing carnivals are often marketed as fun entertainment, with children’s activities, fashion parades, and live music. Similarly, while there has been public commentary related to the normalising impact of sports betting advertising on children, there has been limited community discussion about the harms from sports betting. Tobacco control advocates highlighted the ethical obligation for governments to communicate accurate information about the harms associated with cigarettes, and the actions that can be taken to reduce the harms caused by these products [33]. Although very few mass media campaigns in gambling have taken this approach, the current study shows overwhelming community support for campaigns which focus on educating the community (including children) about the harms associated with gambling products.

Second, this study is consistent with other community surveys in Australia [15, 21, 22] that demonstrate community support for stricter boundaries being placed around gambling products, and the marketing of these products. Support for advertising restrictions in this study is much higher than community support for advertising restrictions relating to other harmful products. For example, a recent survey showed that despite overwhelming evidence on the health and social harms associated with alcohol, and public health advocacy initiatives, only 60% of Australians believed that alcohol should not be promoted at sporting events, and 70% believed that alcohol should not be advertised before 8.30 pm [24]. Similarly, in a survey conducted in 1987 in the early days of opinion polling to assess community support for tobacco controls, only 37% of Victorians supported a ban on tobacco sponsorship of sport [34]. While the Australian government has signalled restrictions on ‘siren to siren’ gambling advertising in sport (up until 8.30 pm at night), these measures fall short of the complete ban supported by participants in this study [35]. Commentators have already observed significant flaws in the proposed restrictions. For example, it is unlikely that children, and in particular teenagers, will turn off their favourite sporting match at 8.30 pm, and as such will still be exposed to gambling advertising. Further, the ban does not close loopholes which allow gambling advertising in ‘G’ rated viewing hours within sports and current affairs programs. Without closing these loopholes, there is a risk that the gambling industry may work with broadcasters to shift advertising to pre-match sports entertainment shows. Nevertheless, it appears that in this instance the Australian government may be paying some attention to community expectations in relation to the promotion of sports betting products. However, questions remain as to why, in the face of community concern and support for regulatory reform, governments have done relatively little to implement comprehensive reforms of the gambling industry that are required to prevent and reduce gambling harm. It has been proposed that such inaction may be due largely to the influence of the gambling industry over government decision-making—via lobbying, political donations, partnerships with policy-makers, and influences on research agendas [1, 36–40]. The current results suggest that existing government approaches to gambling in Australia are out of line with community attitudes to many forms of gambling (such as EGMs), and public expectations of mechanisms for protecting communities from gambling products that may be harmful for communities (for example, through comprehensive restrictions on advertising during sport).

Finally, we would sound a note of caution. History has shown that when there is significant support for the regulation of products, and negative community attitudes towards industries such as gambling, alcohol, and tobacco, those industries may put more strategic effort into counter-measures such as framing themselves as ‘good corporate citizens’ as part of their efforts to avoid or minimise the impact of restrictions and regulations. For example, when there was widespread recognition that cigarettes were lethal, and there was significant community support for strong regulatory measures associated with tobacco products and advertising, tobacco companies put significant resources into reframing themselves as responsible corporate citizens. Strategies included support for charities, youth smoking prevention programs, responsibility advertising, so called self-regulation of advertising, and sponsorship of sport and the arts [41], complemented by heavy lobbying and political donations. We have recently seen similar strategies emerge from the gambling industry. For example, the EGM industry has developed policies advocating for industry led responses to problem gambling, including education strategies for children which focus on teaching them about ‘responsible gambling’ rather than the harms associated with products [42], as well as significant donations to political parties and politicians [43]. More recently, coalitions for corporate bookmakers have stated that they support a reduction in wagering advertising within sport to address the level of ‘angst’ about gambling advertising in the Australian community [44]. They have also established ‘codes of conduct’ which state that they will advocate for reforms (such as prohibiting credit betting) which have already been announced as imminent regulatory measures by government, and have signalled a commitment to fund independent research regarding the prevalence and impacts of online wagering in Australia [45]. As was demonstrated in relation to government proposals to introduce mandatory pre-commitment on EGMs, the gambling industry has already succeeded in lobbying and pressuring politicians to oppose some reforms [46]. It will be important for public health advocates and coalitions to recognise such strategies and to develop new ways of responding to industry efforts to resist reform.

It is important to acknowledge the limitations of this study. First, while we collected data from a sample of participants representative of the Victorian community by age and gender, the sample was skewed towards those who had higher levels of education and lived in more affluent areas of Victoria. As such, it may not be generalisable to the views of the entire Victorian community. Second, the online panel approach to data collection resulted in a sample that was over representative of gamblers and individuals experiencing gambling harm, although the outcomes of the study were still commensurate with the findings from community-based surveys. While online panel studies of gambling behaviour consistently capture populations that gamble more frequently across the full spectrum of gambling harm, there has been limited explanation of why this may occur [26]. Some explanations could be that online surveys afford more anonymity than telephone surveys, and people are likely to more honestly report their gambling behaviours; that online surveys are more effective at recruiting younger adults (and in particular younger men) who gamble online and who may not participate in surveys which focus predominantly on landline-based samples; and that people who have problems with gambling may sign up for online survey companies to take advantage of the incentives offered by these companies. Given the increasing use of online panels by academic researchers as a cost effective way of collecting data, researchers should seek to explain why these differences in samples may be occurring, and the potential impact of either under or over representation biases for evidenced-based policy-making decisions. Third, this initial examination of the study data aimed to present the broad responses from the sample, and did not aim to investigate differences according to age, gender, or gambling characteristics. This is an important area for future research. Fourth, it is important to remember that Victoria does not have the same historical associations with EGMs in clubs, as is found in other states in Australia such as New South Wales. As such, the views of Victorians may not be reflective of individuals in other states and territories. It is important to note that regulatory initiatives were presented to participants without any counter arguments. Typically, the gambling industry has gone to great lengths to oppose increased regulations of their products or their marketing tactics and to create doubt about the effectiveness of potential harm minimisation measures. Coordinated responses to these tactics will be important in ensuring that governments implement harm reduction strategies based on independent evidence rather than industry driven arguments.

## Conclusions

This study indicates that there is a high perception of harm associated with a range of gambling products. While state and federal governments have been reluctant to implement a comprehensive approach to reducing gambling harm, this study indicates that there is significant community support for a range of specific harm reduction and prevention measures. This provides important support for the work of those seeking more effective action to reduce the harms associated with gambling promotions and products.

## Abbreviations

ABS: Australian Bureau of Statistics
ATGS: Attitudes Towards Gambling Scale
EGM: Electronic Gambling Machine
PGSI: Problem Gambling Severity Index
SEIFA: Socio-Economic Indexes for Areas
